# Synthesis and adsorption performance of functionalized chitosan and carboxyethylsilanetriol hybrids

**DOI:** 10.1186/s13065-023-00943-0

**Published:** 2023-04-07

**Authors:** Ahmed Salama, Mohamed El-Sakhawy

**Affiliations:** Cellulose and Paper Department, National Research Centre, 33 El-Bohouth St., Dokki, Giza, 12622 Egypt

**Keywords:** Chitosan, Silica, Hybrid, Adsorption, Water treatment

## Abstract

A novel adsorbent from cationic chitosan derivative and anionic silica precursor was fabricated to remove methylene blue (MB). The hybrid material was prepared from N-guanidinium chitosan acetate (GChi) and carboxyethylsilanetriol sodium salt by a simple ionic interaction followed by sol–gel approach. Multiple characterization methods were used to analyze the morphology and the structure of the well-prepared functionalized material. Batch experiments were conducted to optimize the various operational parameters. The Langmuir isotherm was used to fit the data, and it predicted monolayer adsorption with a maximum capacity of 334 mg g^−1^. A pseudo-second-order equation fit the adsorption process well. Chitosan/silica hybrids containing carboxylic groups are efficient and cost-effective adsorbents for cationic dyes adsorption from aqueous solutions.

## Introduction

Several industries have discharged organic pollutants and heavy metals into surface and ground water in recent years, causing serious water contamination problems [1, 2]. Because dyes are persistent and non-biodegradable, dyes are one of the most harmful pollutants in wastewater. A particular focus has been placed on treating aqueous effluents containing organic dyes [3, 4]. The goal of reducing water pollution has been achieved through various approaches for the purification of effluents. Among them the adsorption process is economically advantageous and efficient, especially when applied to highly diluted aqueous solutions [4, 5]. Environmentally friendly and high-performance water treatment materials have attracted significant industrial and academic attention [6, 7].

Polysaccharides-based composite materials have potential uses in water purification for the elimination of toxic metals and dyes [8–10]. Chitosan was previously viewed as a potential candidate for adsorption in decontamination processes due to its sustainability and biocompatibility [11]. The disadvantages of chitosan include its poor mechanical properties, small specific surface area, and ease of dissolution in acidic solutions. Various wastewater treatment processes use modified chitosan that has been cross-linked [12], or incorporated with magnetic [13] or nanomaterials [6]. For example, by irradiating chitosan, poly (2-acrylamide-2-methyl-1-propanesulfonic acid), and acrylamide, a hydrogel was prepared for adsorption of methylene blue. This hydrogel exhibits both high strength and adsorption efficiency [14]. The free radical polymerization in one pot technique was used by Sethi et al. to prepare an acrylamide grafted chitosan hydrogel for heavy metals adsorption [15]. Efficient adsorbent nanofibers were prepared by electrospinning chitosan with cellulose in a cosolvent system [16]. The adsorption capacity of a hydrogel based on chitosan will therefore be enhanced by increasing the density of new function groups such as amino and carboxylic groups. Chitosan/silica hybrids are attracting substantial attention as an original group of materials with anticipated properties in a wide range of applications such as adsorption [17], drug delivery [18, 19] and bone replacement [20]. Organic dyes can be removed effectively with adsorbents containing carboxylic groups due to their ability to form anionic interactions with cationic groups as well as their ability to form hydrogen bonds. In chemical complexation, the carboxyl and hydroxyl groups have specific site interactions with hazardous metal ions and organic dyes [21].

Guanidinium, a positively charged organic group, is a functional group of vital amino acids. This functional group can interact with an extensive range of anionic groups through ion pairing [22]. Despite this, robust and versatile guanylation of polysaccharides remains a challenge. In terms of natural polymer chemistry, effective functionalization of chitosan with guanidinium groups would be a vital improvement [23].

In the current study, a mild acidic environment was used to achieve guanylation of chitosan using cyanamide in the presence of scandium (III) triflate as a catalyst. Cationic chitosan derivative, GChi, was prepared and chemically crosslinked with carboxyethylsilanetriol sodium salt to form a novel hybrid material. Using guanidinium chitosan/silica containing carboxylic groups, a novel system for sequestering cationic dyes from aqueous solutions was investigated. The adsorption mechanism was evaluated by adsorption kinetics and adsorption isothermal models, and the effects of pH on the adsorption efficiency were calculated. The composite containing carboxylic acid groups and GChi exhibited homogenous and smooth structure to serve as an economic and effective hybrid for the sequestration of cationic dyes.

### Experimental part

#### Materials

Medium molecular weight chitosan (degree of deacetylation, 75–85%), cyanamide and scandium (III) triflate were purchased from Sigma-Aldrich. Carboxyethylsilanetriol sodium salt (25% in water) was provided from abcr GmbH. All chemicals were of analytical grade and used without further purification.

### Syntheses

#### GChi preparation

In this experiment, GChi was prepared according to our previous work [24]. In a round flask, 10 ml of 2% chitosan dissolved in acetic acid was mixed with 84 mg of cyanamide (0.002 mol) and 30 mg (60 nmol) of scandium (III) triflate. After stirring at 100 °C for 48 h, the formed chitosan derivative was precipitated and frequently cleaned with acetone.

#### GChi/silica containing carboxylic groups

In two steps, GChi/silica composite was synthesized. A suspension of 0.4 g GChi in 100 ml of deionized water was treated with two equivalents of carboxyethylsilanetriol sodium salt. As a next step, 0.02 M HCl solution (10 mL) was added dropwise into the reaction mixture, then the solution was heated at 60 °C for 48 h. The formed white precipitate was then washed with deionized water until neutral pH, followed by methanol washing and freeze drying.

### Characterization methods

Elemental Analysis was performed with an Elemental Vario Micro Cube apparatus. Solid State NMR Spectroscopy ^13^C cross polarization magic angle spinning (CP/MAS) was utilized to achieve a high signal-to-noise ratio with 5 ms contact time and 5 s recycling delay. The attenuated total reflectance Fourier transform infrared spectra were carried out utilizing Thermo Nicolet FT-IR Avatar 320 with a diamond crystal Spectra. TGA measurements were carried out with a NETZSCH STA 409 PC instrument at a heating rate of 5 °C/min. SEM Analysis The surface morphology of the hybrid was determined by using a Hitachi S-4800 scanning electron microscope.

### Batch adsorption studies

MB solutions were prepared by diluting stock solution (1000 ppm) with water to create concentrations of 50–600 ppm. The dye solution concentrations were measured by spectrophotometer (UNICO UV-2000) at 670 nm maximum absorbance. Using a sample adsorption technique, 50 mg of dried adsorbent was placed in glass bottles containing 50 ml of MB solution. At 25 °C, stirring of the solutions was done at various intervals after adding MB. The pH of the dye solutions was adjusted with 0.1 M HCl or 0.1 M NaOH aqueous solutions to study the effects of pH. The amount of adsorbed dye at adsorption equilibrium, *q*_*e*_ (mg/g), was calculated from Eq. (1):

1 $$q_{e} = \,\frac{{\left( {co - ce} \right)V}}{W}$$

where C_o_ and C_e_ are the initial and equilibrium dye concentrations (mg/L), V is the volume (L) of the dye, and W is the weight of the composite (g). Data are representative of at least three experiments.

### MB desorption

GChi/silica hybrids were tested for regeneration using 0.5 M HCl as an eluent. The composite loaded with MB was agitated with 10 mL acidic solution for 12 h. A spectrophotometric method was used to determine the final MB concentration.

### pH point of zero charge(pHzpc) analysis

The pH at point of zero charge (pHpzc) is an important parameter to get a better understanding of the surface adsorption mechanism. The pHzpc of the adsorbent was carried out using the simple solid addition method [25]. In brief, a solution of 0.1 mol/L NaCl was prepared and boiled to remove dissolved CO_2_ and then cooled to room temperature. Initial pH (pHi) of this solution was adjusted from pH 2 to 12 by adding either 0.1 mol/L HCl or 0.1 mol/L NaOH. Adsorbent dose (0.1 g) was added to 50 mL of 0.1 mol/L NaCl solution in 100 mL conical flasks and stirred in a shaker at 150 rpm and 25 °C for 300 min and then, final pH (pHf) of solution was measured. The graph was plotted between the difference between the final and initial pH (pHf-pHi) against the initial pH (pHi) and the point at which pHf-pHi = 0 was taken as the pHzpc of the adsorbents.

## Results and discussion

### GChi/silica composite characterization

**Fig. 1:**
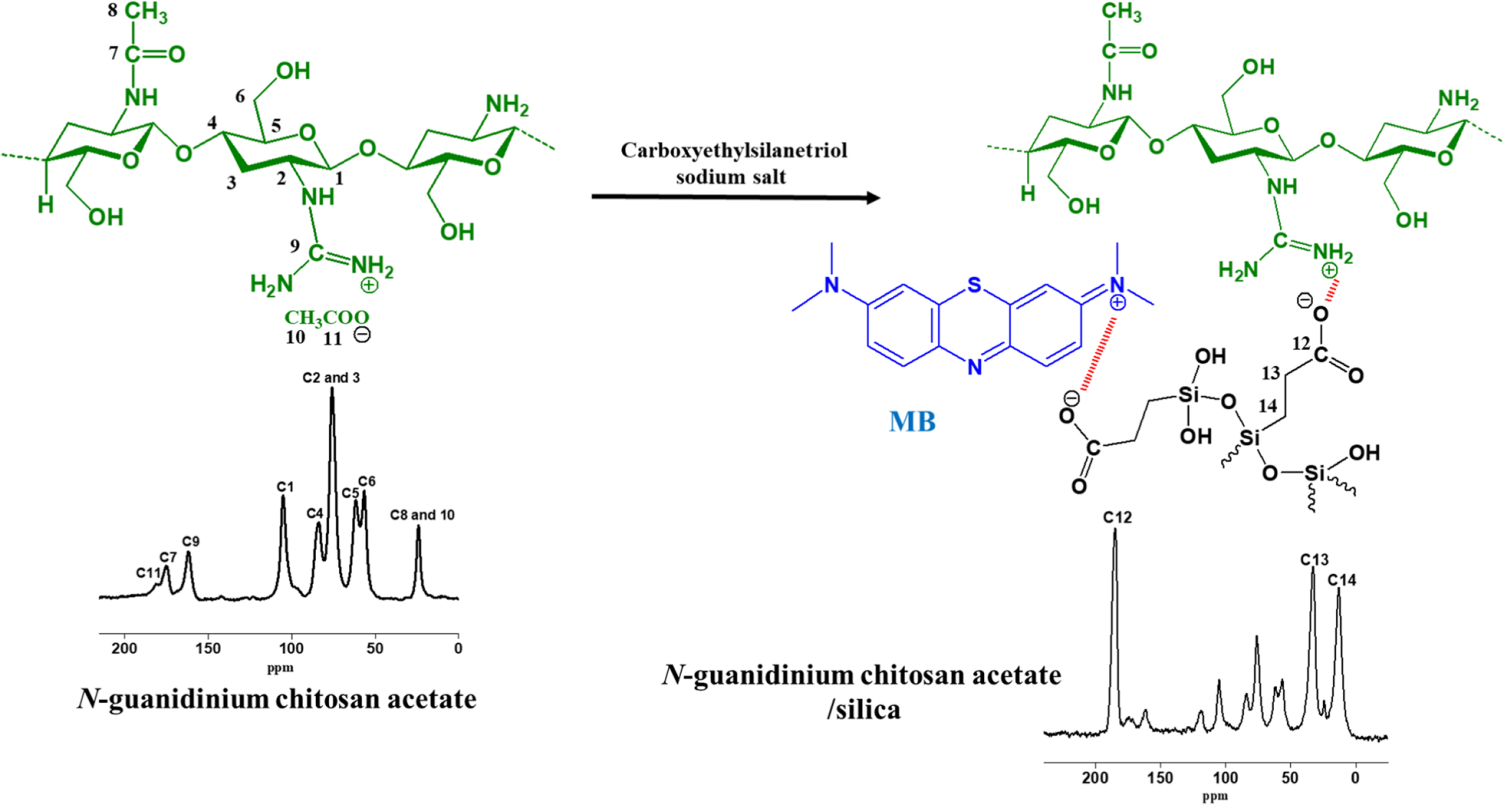
Structure and ^13^C CP-MAS of cationic chitosan derivative, the hybrid containing carboxylic group and MB structure

As reported in our previous study, GChi was prepared by a direct interaction between chitosan and cyanamide [24]. The hybridization was made by reacting chitosan containing guanidinium groups with sodium carboxyethylsilanetriol followed by silanol condensation. Elemental analysis revealed that chitosan contains 7.7% nitrogen and GChi contains 11.1% nitrogen. The chemical structure of the obtained material via the reaction of cationic chitosan derivative with silica precursor is shown in Fig. 1.

Figure 1 shows ^13^C CP-MAS solid state NMR experiments and the carbon signals are labeled according to the illustrated structure. Anhydroglucose signals were observed in the range of 55–110 ppm for chitosan derivative and corresponding composite. The peak of GChi, which is attributed to the guanidinium group, occurred at 160 ppm. Moreover, acetate counter ions contribute an additional peak at 180 ppm. Additional signals at 13–32 ppm and 185 ppm seen in the spectrum of N-guanidinium chitosan/silica containing carboxylic groups refer to the carbon centers of the ethyl chain (C13/C14) and carboxylate groups (C12).

**Fig. 2:**
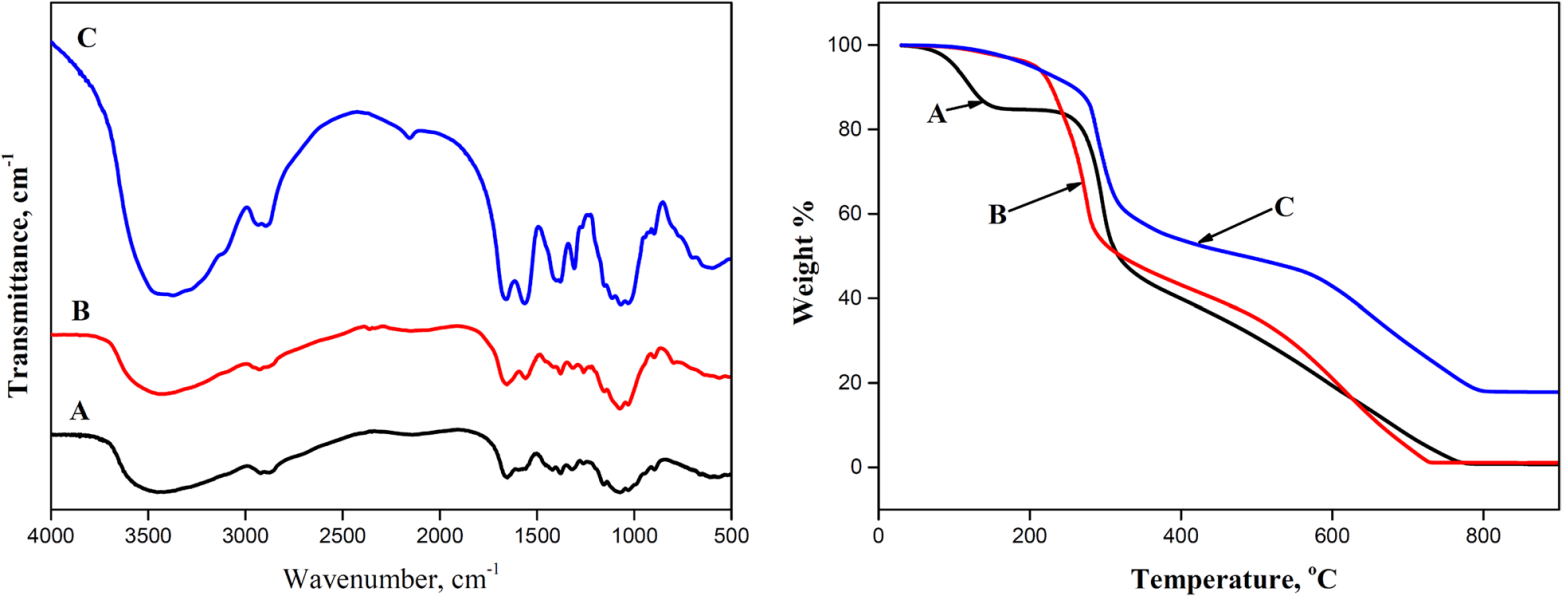
FT-IR spectra and TGA of chitosan **A**, GChi **B** and the formed chitosan/silica hybrid containing carboxylic groups **C**

FT-IR spectra of chitosan, cationic chitosan derivative, and GChi/silica hybrid are shown in Fig. 2. Characteristic band around 3450 cm^−1^ that are attributed to NH_2_ and OH stretching vibrations is clear in all charts. The peaks at 1657 and 1579 cm^−1^ correspond to the stretching vibrations of C = O and NH, respectively. N-guanidinium chitosan shows a strong adsorption band at 1629 cm^−1^, indicating the presence of guanidinium moiety [26]. A carboxyethylsilanetriol moiety is responsible for the two bands at 2939 and 2847 cm^−1^. Additionally, a pronounced absorption peak between 1119 and 1066 cm^−1^ attributed to the Si–O–Si bonds was observed [27]. Chitosan/silica composite exhibits more intense and broader bands around 3400 cm^−1^, which can be attributed to the presence of residual silanol groups.

**Fig. 3:**
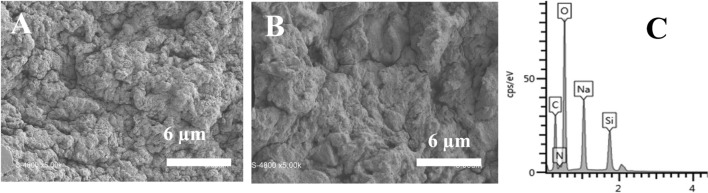
SEM of GChi **A** and GChi/silica composite **B** and EDX for the hybrid material **C**

A thermogravimetric study was conducted on chitosan, GChi and GChi /silica hybrid to investigate silica content and the effect of silica on thermal stability. As shown in Fig. 3, the TGA thermograms for the three materials show three main regions of mass loss. For neat chitosan, the initial weight loss was high (approximately 15%), which was observed below 200 °C, likely due to physiosorbed water evaporating. The weight loss for GChi and the GChi/silica hybrid was the same (4%) indicating their low hydrophilic properties when compared to neat chitosan. Thermal degradation of hydrocarbon backbone of chitosan and organic material in the silica precursor is the reason for weight loss during region II (200–450 °C). However, GChi degrades at a lower temperature (~ 220 °C) than chitosan (~ 260 °C) and GChi/silica composite (~ 275 °C). In region II, N GChi, chitosan, and GChi /silica hybrid lost 58, 55, and 45 percent of their weight, respectively. It was observed at 770 °C that a complete mass loss had occurred in region III. Furthermore, the GChi/silica exhibits greater thermal stability than chitosan and GChi. As a result, the remaining mass of the hybrid is 18%, demonstrating that GChi/silica hybrid holds silica content of 18%.

SEM was used to examine the morphology of the cationic chitosan and the hybrid containing carboxylic acid. Figure 3A shows the surface of GChi, which is relatively rough without retaining any distinct shape. Conversely, the hybrid containing carboxylic acid showed smooth surface consisting of agglomerated microparticles (Fig. 3B). The smooth surface of the hybrid reflects the homogeneity between inorganic silica and GChi. The energy dispersive X-ray pattern (Fig. 3C) shows that the hybrid is primarily composed of silicon (Si), carbon (C), oxygen (O), nitrogen (N), and sodium (Na). GChi generates carbon and nitrogen, while silicon and sodium come from silica.

### Application of the GChi /silica composite for MB adsorption

Different factors, such as adsorbent dosage, pH, equilibrium time, and primary dye concentration, were evaluated to determine whether the current GChi/silica containing carboxylic groups is capable of MB adsorption.

### Effect of adsorbent dosage

**Fig. 4:**
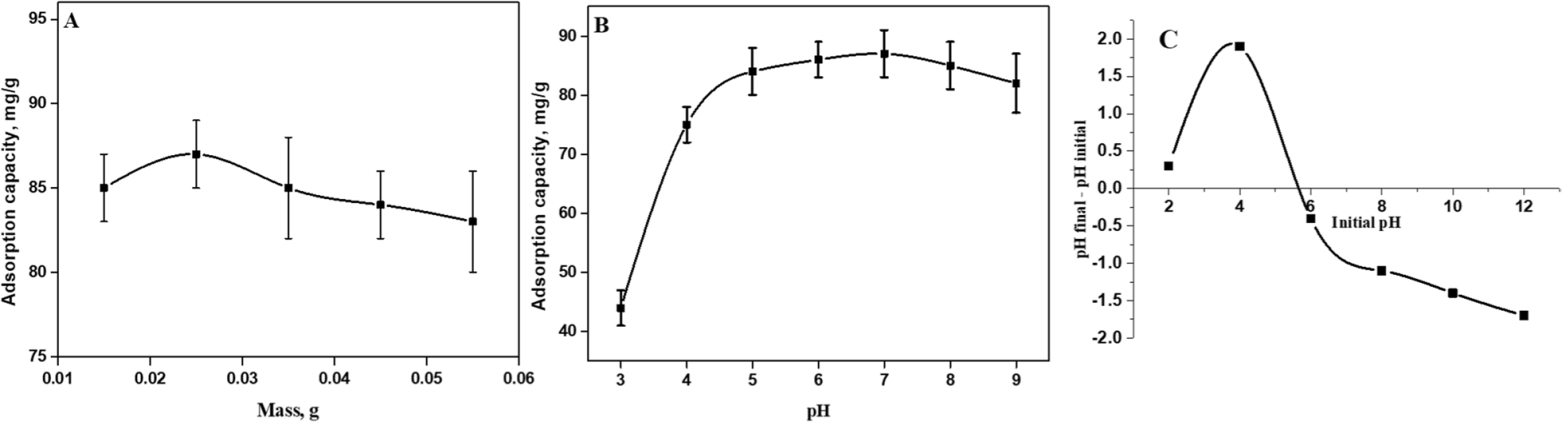
**A** Effect of adsorbent mass (initial of dye concentration = 100 mg/L, pH = 7, and time = 90 min), **B** Effect of pH on adsorption (initial of dye concentration = 100 mg/L, dosage of GChi/silica microspheres = 0.025 g, time = 90 min) and **C** pH zero-point charge of GChi/silica containing carboxylic groups

Variation of MB adsorption with the hybrid dosage is described in Fig. 4A in the range of 0.015–0.055 g of dried hybrid (the initial MB concentration is 100 mg/L). The maximum adsorption capacity reached the maximum adsorption at 0.025 g of adsorbent. As hybrid amounts increased, more unsaturated adsorptive sites remain active, which reduced adsorption capacity. Further experiments were performed with a fixed dose of 0.025 g of dried adsorbent.

#### Effect of pH on MB adsorption

Adsorption is influenced significantly by the pH of a dye solution [28]. As part of the experiments, dye solutions (with a known concentration of 100 mg L^−1^) were prepared, and their pH was varied between 3 and 9. A rapid growth in adsorption capacity was detected with a pH increase to record 84 mg/g at pH 5. As presented in Fig. 4, the maximum removal capacity was obtained at pH 7 (87 mg/g). Lower pH values will produce more H^+^ that competes strongly with cationic dye for adsorption on the active positions on the composite surface. Additionally, by lowering the acidity of the aqueous solution below 2, the hybrid surface becomes negatively charged. The charge on the COO**¯** functional group promotes ionic interactions with positively charged dye molecules, and therefore, the adsorption capacity of the ionic composite material in an acid solution is lower due to a reduction in anionic adsorption sites.

The pH at zero-point charge (pHzpc) of the adsorbent is an important parameter to determine under what pH adsorbent has positive or negative surface charge. At pHzpc the surface of adsorbent is neutral. Below this value, the surface is positively charged; beyond this value, it is negatively charged. The ionizable functional groups, such as NH_2_ or OH, on the surface of chitosan, may gain or lose a proton, resulting in a surface charge that varies with pH. To better evaluation of the pH effect on adsorption, the pHzpc that can describe the surface charge properties of materials was measured. The values for the pHzpc of the adsorbent composite were obtained at 5.63, (Fig. 4C). The surface of the adsorbent may become positively charged with H^+^ ions below the pHzpc leading to electrostatic repulsions with the cationic MB. While optimum adsorption occurs at pH above pHzpc because binding groups such as amine, carboxyl and hydroxyl lose their protons and the surface becomes negatively charged favoring MB removal and the hybrid between MB molecules on the adsorbent surface is less.

#### Effect of contact time

**Fig. 5:**
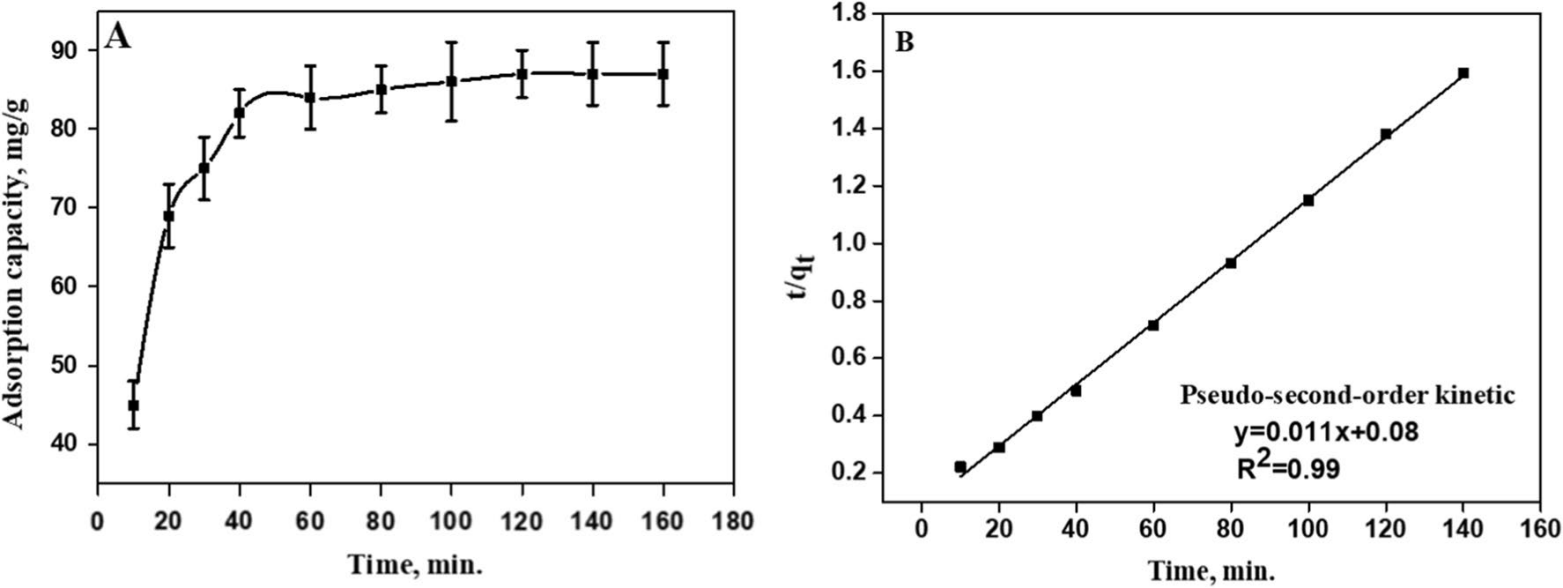
**A** Adsorption capacity of MB by GChi/silica containing carboxylic at different time intervals (Initial of dye concentration = 100 mg/L, pH = 7) and **B** the fitting of Pseudo-second order kinetic

The adsorption systems having initial dye concentration of 100 mg L^−1^ and 25 mg of adsorbent were kept in contact for a time range of 10 to 160 min with constant stirring. As the contact time increased, the adsorption increased up to 82 mg/g. A period of slower adsorption capacity increase took place between ~ 40 and ~ 80 min following this interval. In the end, the adsorption plateaued after 90 min. A contact time of 90 min is sufficient to achieve equilibrium of the adsorption, as shown in Fig. 5.

##### Adsorption kinetics

During adsorption process, dye molecules are transferred from the liquid phase to the adsorption surface. When dye molecules migrate to the outer surface of the hybrid containing carboxylic, they diffuse through the inner sites. MB adsorption was explored using two commonly used kinetic models, namely pseudo-first-order and pseudo-second-order. The linear pseudo-first order (Eq. 2) and pseudo-second-order (Eq. 3) are shown in Eqs. 2 and 3.

2 $$\log \left( {q_{e } - q_{t } } \right) = \log (q_{e } ) - \,\frac{{k_{1} }}{2.303}\,t$$

3 $$\frac{t}{{q_{t} }}\, = \,\frac{t}{{q_{e} }}\, + \,\frac{1}{{k_{2} q_{e}^{2} }}$$

Adsorption capacity at time t (minute), and adsorption capacity at equilibrium (mg/g) are given by q_t_ (mg/g) and q_e_ (mg/g). The pseudo-first order and second order kinetics rate constants are k1 (min ^−1^) and k_2_ (g mg^−1^ min^−1^). According to the data in Table 1 (R^2^ and χ^2^), the pseudo-second-order model can be proposed for adsorption kinetics, depending on the amount of solute adsorbed on the surface of adsorbent and the amount of solute adsorbed at equilibrium. Compared to the pseudo-first-order model, which offered a correlation coefficient of 0.78 and χ^2^ 33.6, the pseudo-second-order model provides better fit to the experimental data. The value of *q*_*e,cal*_ from the pseudo-second-order kinetic model is close to experimental value than the ones obtained from the pseudo-first-order value which suggests the chemical adsorption occurrence.

**Table 1:** Kinetic parameters for MB adsorption by the hybrid containing carboxylic acid group

Pseudo first order-model					Pseudo second- order model			
q_e, exp_ (mg/g)	q_e,cal_ (mg/g)	K_1_(min^−1^)	R^2^	χ^2^	q_e, cal_ (mg/g)	K_2_ (g mg^−1^ min^−1^)	R^2^	χ^2^
87	31	0.013	0.78	33.6	91	15.1 × 10^–4^	0.998	3.4

#### Adsorption isotherm

**Fig. 6:**
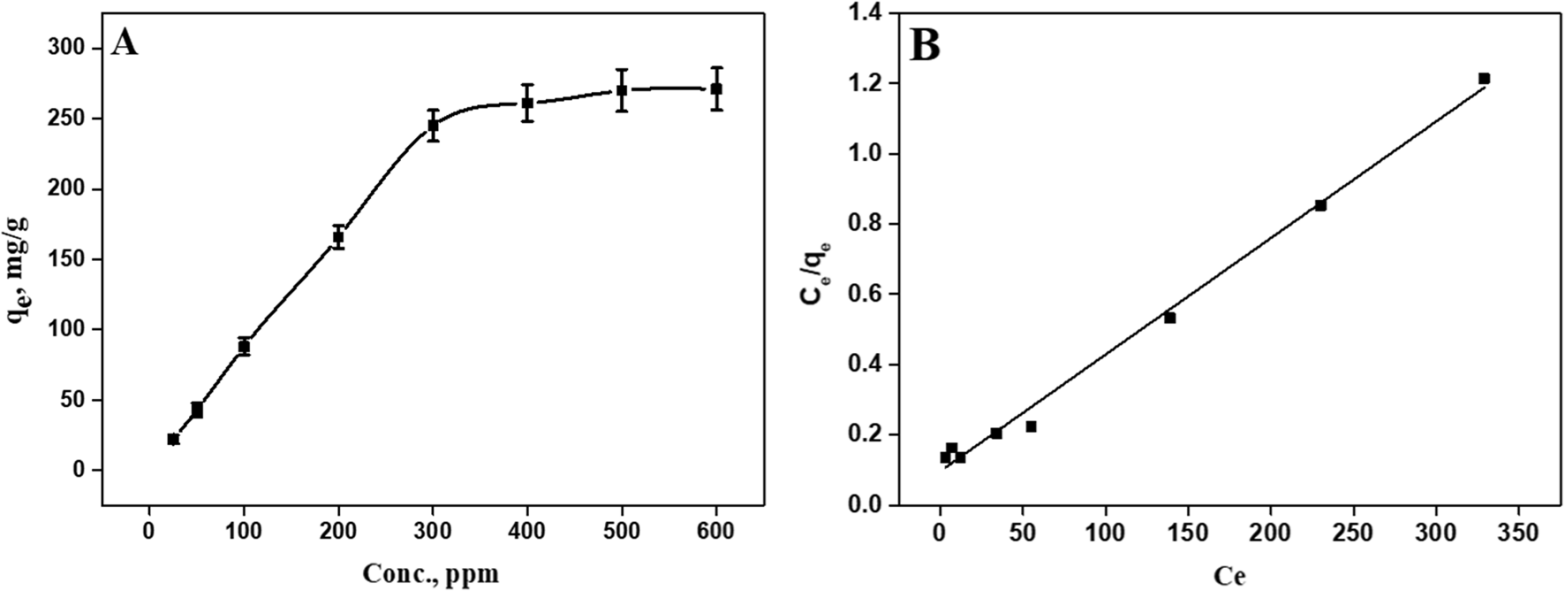
**A** Effect of MB initial concentration on the adsorption capacity of the current hybrid and **B **the fitted Langmuir isotherm model

Adsorption isotherms are essential for evaluating the adsorption mechanism of MB on sorbent surfaces. In Fig. 6, the initial MB concentration and the equilibrium adsorption are shown. Clearly, the equilibrium adsorption increases with an increase in MB concentration. Adsorption capacity of the hybrids increases rapidly until a plateau is reached. Transfer of MB molecules from solution to the surface of the hybrid is primarily driven by the initial concentration of MB molecules.

Models such as the Langmuir and Freundlich are used to simulate the adsorption isotherm. The Langmuir isotherm model is applicable to specific homogeneous sorption processes with similar activation energies between sorbate molecules. The Langmuir model (Eq 4) is linearized as follows [29, 30]:

4 $$\frac{{C_{e} }}{{q_{e} }} = \frac{{K_{s} }}{{q_{\max } }} + \frac{{C_{e} }}{{q_{\max } }}$$

where *q*_e_ is the equilibrium adsorption (mg/g), *C*_e_ is the equilibrium concentrations in the solution (mg/l), *q*_max_ represents the maximum adsorption (mg/g), and *K*_s_ is a constant of the Langmuir model (mg/l). To evaluate the best-fit model, we used chi-squared error tests (χ^2^) and linear regression coefficients (R^2^). It is more likely that the experiment and the model are aligned with the smaller χ^2^ value.

The linearized Langmuir equation exhibited a correlation coefficient near 1, according to Table 2. These results propose that MB adsorption is described exactly by the Langmuir model. According to Langmuir plot, the values of *q*_*max*_ and *K*_s_ are 935 mg/g and 36.7 mg/l, respectively. The Langmuir isotherm assumes an adsorption site with equal energies and enthalpies in a monolayer. Consequently, the current hybrid material shows homogenous nature of the adsorption sites.

5 $$\log {\text{q}}_{{\text{e}}} = \frac{1}{{\text{n}}}\log {\text{C}}_{{\text{e}}} + \log {\text{K}}_{{\text{f}}}$$

**Table 2:** Parameters for MB adsorption by the hybrid containing carboxylic acid group according to various models

Langmuir isotherm constants				Freundlich isotherm constants			
K_s (_mg/L)	q_m_(mg/g)	R^2^	χ^2^	K_F_ (mg g^−1^)/(gL^−1^)^1/n^	n	R^2^	χ^2^
32.3	334	0.99	5.9	18	1.88	0.86	22.3

Freundlich model (Eq. 5), which is based on adsorption on heterogeneous surface, exhibited that the linear coefficient was 0.928. The values of the Freundlich constants *K*_*F*_ and *n* are 51 and 2.13 respectively (as shown in Table 2).

### Adsorption mechanism

The adsorption of MB by GChi/silica hybrid has been confirmed as monolayer chemical adsorption, and the adsorption process was favorable. GChi/silica hybrid surface was negatively charged due to protonation of carboxylate groups. Additionally, MB was ionized in the aqueous solution and positively charged because of the presence of tertiary amines. During the adsorption process, the GChi /silica interacted with methylene blue through electrostatic attraction. Consequently, this kind of synergistic adsorption enhanced the adsorption efficiency of MB. Moreover, the adsorption process is thought to be mainly driven by electrostatic attraction interactions. Besides, the surface of GChi/silica carboxylate holds many hydroxyls and guanidinium groups, and these functional groups may be combined with the N atom on MB, causing dipole–dipole H-bonding interactions which enables the adsorption procedure. Furthermore, the opportunity of n–π stacking interaction may be proposed. The n–π stacking interaction frequently happened from the lone pair of electrons on an oxygen atom to the π orbital of the aromatic ring of MB [31]. Thus, there may be n–π stacking interactions in this case as well.

### Reusability

**Fig. 7:**
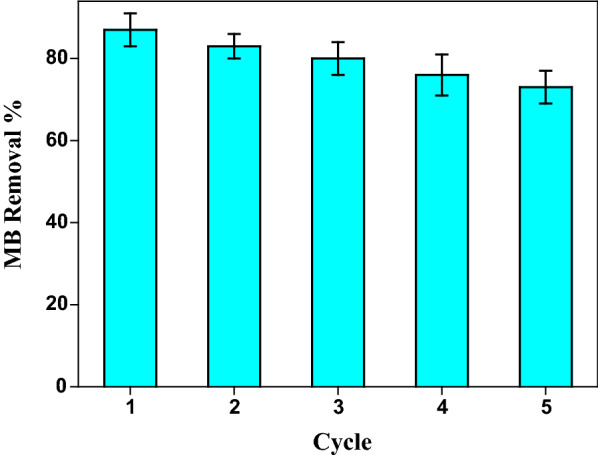
Removal efficiency of MB by GChi/silica hybrid

Practical applications of adsorbents require some degree of reusability and regeneration. To investigate the regeneration performance of the GChi/silica hybrid, we calculated adsorption–desorption cycle experiments. MB was adsorbed onto the hybrid and then desorbed using hydrochloric acid. Adsorption–desorption cycle schematic is shown in Fig. 7. There was a decrease in removal efficiency from 89 to 73% after five cycles. In conclusion, GChi/silica hybrid obviously can be regenerated and applied as a useful adsorbent for eliminating organic dyes. A summary of the adsorption capacities of chitosan based adsorbents towards MB is presented in Table 3. According to these results, GChi/silica hybrids are a promising material for MB absorption due to their higher adsorption capacities than other chitosan-based adsorbents.

**Table 3:** Adsorption capacities of chitosan-based hybrids for MO adsorption

Adsorbent	Conditions	Maximum adsorption (mg/g)	References
Chitosan/silica nanocomposite	Adsorbent dosage = 0.5 g/L, pH = 7, time = 60 min. and MB concentration = 900 ppm	848	[32]
N-guanidinium chitosan/silica hybrid	Adsorbent dosage = 0.5 g/L, pH = 7, time = 90 min. and MB concentration = 600 ppm	334	here
Chitosan/silica/ZnO nanocomposite	Adsorbent dosage = 0.5 g/L, pH = 7, time = 70 min. and MB concentration = 550 ppm	293	[33]
Chitosan/Azadirachta indica leaves/Kaolinite nanocomposite	Adsorbent dosage = 0.02 g/L, pH = 7, equilibrium time = 240 min. and MB concentration = 100 ppm	224	[34]
Chitosan–montmorillonite/polyaniline nanocomposite	Adsorbent dosage = 0.025 g, time = 120 min., and MB concentration = 20 ppm	111	[35]
Graphene oxide/chitosan composite aerogel	Adsorbent dosage = 0.8 g/L, pH = 11, time = 80 min. and MB concentration = 200 ppm	110	[36]

## Conclusion

In the current study, GChi, was prepared and characterized as new cationic chitosan derivative. To improve the adsorption capacity, cationic chitosan derivative was chemically modified with anionic silica precursor followed by sol–gel reaction to form hybrid material. The SEM images of ionic GChi/silica demonstrates that the hybrid has significant degree of homogeneity between chitosan derivative and silica precursor and the residual silica recorded 18%. Also, the images of the hybrids revealed rough surface, providing more adsorption sites for dye adsorption. The maximum adsorption capacity of the hybrid was calculated by fitting the Langmuir equation to the adsorption isotherm and was found to be 334 mg/g. The removal efficiency of MB is 73% after five adsorption–desorption cycles. The high adsorption capacity attributes to the ionic nature of the prepared hybrid. As an adsorbent for cationic pollutants from aqueous solutions, as well as an alternative bioadsorbent to replace conventionally expensive analogs, the current material is promising.

## Data Availability

The datasets used and/or analyzed during the current study are available from the corresponding author on reasonable request.
